# Supplementary material for: Pyruvate Dehydrogenase Kinase 1 inhibition mediated oxidative phosphorylation enhancement in cartilage promotes osteoarthritis progression
Source: BMC Musculoskelet Disord. 2023 Jul 20;24:597. doi: 10.1186/s12891-023-06585-6 (PMC10357736; doi:10.1186/s12891-023-06585-6)
Supplement: Supplementary file 1 — Additional file 1. [file 12891_2023_6585_MOESM1_ESM.zip › BP.Gsea.1653623667859/GOBP_APOPTOTIC_PROCESS_INVOLVED_IN_MORPHOGENESIS.html]

Details for gene set GOBP\_APOPTOTIC\_PROCESS\_INVOLVED\_IN\_MORPHOGENESIS[GSEA]

|  || Dataset | 55457\_collapsed\_to\_symbols.cls.cls #OA\_versus\_normal.cls.cls #OA\_versus\_normal\_repos |
| Phenotype | cls.cls#OA\_versus\_normal\_repos |
| Upregulated in class | normal |
| GeneSet | GOBP\_APOPTOTIC\_PROCESS\_INVOLVED\_IN\_MORPHOGENESIS |
| Enrichment Score (ES) | -0.51834404 |
| Normalized Enrichment Score (NES) | -1.730405 |
| Nominal p-value | 0.003502627 |
| FDR q-value | 0.12143461 |
| FWER p-Value | 1.0 |
Table: GSEA Results Summary

  

Fig 1: Enrichment plot: GOBP\_APOPTOTIC\_PROCESS\_INVOLVED\_IN\_MORPHOGENESIS      
 Profile of the Running ES Score & Positions of GeneSet Members on the Rank Ordered List

  

| SYMBOL | TITLE | RANK IN GENE LIST | RANK METRIC SCORE | RUNNING ES | CORE ENRICHMENT || 1 | VDR | vitamin D receptor [Source:HGNC Symbol;Acc:HGNC:12679] | 3571 | 0.197 | -0.2525 | No |
| 2 | PAX8 | paired box 8 [Source:HGNC Symbol;Acc:HGNC:8622] | 3680 | 0.187 | -0.2371 | No |
| 3 | LRP5 | LDL receptor related protein 5 [Source:HGNC Symbol;Acc:HGNC:6697] | 4305 | 0.128 | -0.2693 | No |
| 4 | CRYAB | crystallin alpha B [Source:HGNC Symbol;Acc:HGNC:2389] | 4742 | 0.092 | -0.2915 | No |
| 5 | FOXC1 | forkhead box C1 [Source:HGNC Symbol;Acc:HGNC:3800] | 5227 | 0.054 | -0.3223 | No |
| 6 | FZD5 | frizzled class receptor 5 [Source:HGNC Symbol;Acc:HGNC:4043] | 5392 | 0.041 | -0.3298 | No |
| 7 | PAX2 | paired box 2 [Source:HGNC Symbol;Acc:HGNC:8616] | 6564 | -0.047 | -0.4149 | No |
| 8 | TGFB2 | transforming growth factor beta 2 [Source:HGNC Symbol;Acc:HGNC:11768] | 6985 | -0.079 | -0.4375 | No |
| 9 | LEF1 | lymphoid enhancer binding factor 1 [Source:HGNC Symbol;Acc:HGNC:6551] | 7467 | -0.116 | -0.4602 | No |
| 10 | BMP4 | bone morphogenetic protein 4 [Source:HGNC Symbol;Acc:HGNC:1071] | 7816 | -0.142 | -0.4692 | No |
| 11 | TNFRSF1A | TNF receptor superfamily member 1A [Source:HGNC Symbol;Acc:HGNC:11916] | 8196 | -0.171 | -0.4770 | No |
| 12 | HAND2 | heart and neural crest derivatives expressed 2 [Source:HGNC Symbol;Acc:HGNC:4808] | 8211 | -0.172 | -0.4562 | No |
| 13 | SPI1 | Spi-1 proto-oncogene [Source:HGNC Symbol;Acc:HGNC:11241] | 9012 | -0.232 | -0.4889 | Yes |
| 14 | HNF1B | HNF1 homeobox B [Source:HGNC Symbol;Acc:HGNC:11630] | 9072 | -0.237 | -0.4633 | Yes |
| 15 | BAK1 | BCL2 antagonist/killer 1 [Source:HGNC Symbol;Acc:HGNC:949] | 9266 | -0.253 | -0.4463 | Yes |
| 16 | BAX | "BCL2 associated X, apoptosis regulator [Source:HGNC Symbol;Acc:HGNC:959]" | 9323 | -0.257 | -0.4180 | Yes |
| 17 | BMP7 | bone morphogenetic protein 7 [Source:HGNC Symbol;Acc:HGNC:1074] | 9544 | -0.278 | -0.3998 | Yes |
| 18 | NKX2-5 | NK2 homeobox 5 [Source:HGNC Symbol;Acc:HGNC:2488] | 9875 | -0.309 | -0.3863 | Yes |
| 19 | FGF4 | fibroblast growth factor 4 [Source:HGNC Symbol;Acc:HGNC:3682] | 9958 | -0.317 | -0.3524 | Yes |
| 20 | SCRIB | scribble planar cell polarity protein [Source:HGNC Symbol;Acc:HGNC:30377] | 10496 | -0.372 | -0.3469 | Yes |
| 21 | PPP2R1B | protein phosphatase 2 scaffold subunit Abeta [Source:HGNC Symbol;Acc:HGNC:9303] | 10578 | -0.382 | -0.3048 | Yes |
| 22 | TNFRSF1B | TNF receptor superfamily member 1B [Source:HGNC Symbol;Acc:HGNC:11917] | 10969 | -0.427 | -0.2810 | Yes |
| 23 | JAG2 | jagged canonical Notch ligand 2 [Source:HGNC Symbol;Acc:HGNC:6189] | 11039 | -0.435 | -0.2311 | Yes |
| 24 | BCL2L11 | BCL2 like 11 [Source:HGNC Symbol;Acc:HGNC:994] | 11867 | -0.564 | -0.2239 | Yes |
| 25 | FOXC2 | forkhead box C2 [Source:HGNC Symbol;Acc:HGNC:3801] | 12218 | -0.650 | -0.1686 | Yes |
| 26 | NOTCH1 | notch receptor 1 [Source:HGNC Symbol;Acc:HGNC:7881] | 12555 | -0.776 | -0.0963 | Yes |
| 27 | CCN1 | cellular communication network factor 1 [Source:HGNC Symbol;Acc:HGNC:2654] | 12802 | -0.970 | 0.0076 | Yes |
Table: GSEA details [plain text format]

  

Fig 2: GOBP\_APOPTOTIC\_PROCESS\_INVOLVED\_IN\_MORPHOGENESIS      
 Blue-Pink O' Gram in the Space of the Analyzed GeneSet

  

Fig 3: GOBP\_APOPTOTIC\_PROCESS\_INVOLVED\_IN\_MORPHOGENESIS: Random ES distribution      
 Gene set null distribution of ES for **GOBP\_APOPTOTIC\_PROCESS\_INVOLVED\_IN\_MORPHOGENESIS**
